# Genotyping, Assessment of Virulence and Antibacterial Resistance of the Rostov Strain of *Mycobacterium tuberculosis* Attributed to the Central Asia Outbreak Clade

**DOI:** 10.3390/pathogens9050335

**Published:** 2020-04-30

**Authors:** Mikhail V. Fursov, Egor A. Shitikov, Julia A. Bespyatykh, Alexander G. Bogun, Angelina A. Kislichkina, Tatiana I. Kombarova, Tatiana I. Rudnitskaya, Natalia S. Grishenko, Elena A. Ganina, Lubov V. Domotenko, Nadezhda K. Fursova, Vasiliy D. Potapov, Ivan A. Dyatlov

**Affiliations:** 1 State Research Center for Applied Microbiology and Biotechnology, Obolensk 142279, Russia; bogun@obolensk.org (A.G.B.); angelinakislichkina@yandex.ru (A.A.K.); kombarova.tatyana@yandex.ru (T.I.K.); rudnitskaya.tat@yandex.ru (T.I.R.); natalya.grishenko60@mail.ru (N.S.G.); ganin43@yandex.ru (E.A.G.); domotenko@obolensk.org (L.V.D.); n-fursova@yandex.ru (N.K.F.); potapovvd@mail.ru (V.D.P.); dyatlov@obolensk.org (I.A.D.); 2 Federal Research and Clinical Center of Physical-Chemical Medicine, Moscow 119435, Russia; juliabespyatykh@gmail.com

**Keywords:** Beijing genotype, Central Asia Outbreak, murine infection model, Virulence, pre-XDR-TB

## Abstract

The Central Asia Outbreak (CAO) clade is a growing public health problem for Central Asian countries. Members of the clade belong to the narrow branch of the *Mycobacterium tuberculosis* Beijing genotype and are characterized by multidrug resistance and increased transmissibility. The Rostov strain of *M. tuberculosis* isolated in Russia and attributed to the CAO clade based on PCR-assay and whole genome sequencing and the laboratory strain H37Rv were selected to evaluate the virulence on C57Bl/6 mice models by intravenous injection. All mice infected with the Rostov strain succumbed to death within a 48-day period, while more than half of the mice infected by the H37Rv strain survived within a 90-day period. Mice weight analysis revealed irreversible and severe depletion of animals infected with the Rostov strain compared to H37Rv. The histological investigation of lung and liver tissues of mice on the 30th day after injection of mycobacterial bacilli showed that the pattern of pathological changes generated by two strains were different. Moreover, bacterial load in the liver and lungs was higher for the Rostov strain infection. In conclusion, our data demonstrate that the drug-resistant Rostov strain exhibits a highly virulent phenotype which can be partly explained by the CAO-specific mutations.

## 1. Introduction

Worldwide, tuberculosis (TB) is a major public health problem. Despite a slight decrease in TB incidence rates in recent years (1.6% per year in the period 2000−2018 and 2.0% between 2017 and 2018), the situation remains extremely tense. A total of 1.5 million people died from the disease and more than 9 million new cases were detected in 2018 worldwide [1]. Currently, seven lineages of *Mycobacterium tuberculosis* are described, which can cause disease and demonstrate specific phylogeographic patterns [2]. Of them, lineage 2 and lineage 4 are the most widely dispersed, affecting humans across the world. Lineage 2 (or East Asian lineage) is arguably the most widespread and the Beijing genotype family is its major component (13% of global *M. tuberculosis* population; predominant in East Asia and Northern Eurasia) [3,4].

Multiple clinical and epidemiological studies demonstrated a strict association of Beijing genotype members with a high level of drug resistance combined with a large number of compensatory mutations, as well as enhanced pathogenicity, which lead to increased transmissibility and rapid progression of infection [5,6,7]. However, virulence studies provide less conclusive results, showing a variety of phenotypes. The latter is confirmed in animal models [8,9] and in vitro models of macrophage infection [10,11]. This is due to the fact that hypervirulence is not a characteristic feature of the Beijing genotype, but is specific only for certain genetic sublineages, often associated with disease outbreaks in some regions [12,13]. One of the most detailed examples is the spread of the virulent *M. tuberculosis* Beijing B0/W148 cluster in the Russian territory [14].

Phylogenetically, members of lineage 2 may be assigned to at least two large branches, termed ancient and modern sublineages, and 11 populations belong to these branches [15]. Modern Beijing sublineage strains are prevalent worldwide, leading to speculation that this sublineage has hypervirulent features [16]. In the present study, we aimed to investigate a strain belonging to a more homogenous group within modern Beijing called Central Asia Outbreak (CAO) clade. This clade is a part of the Central Asian population. The latter was initially designated as CC1 or Central Asian [6] and then as East Europe 1 [17] and Central Asian and Russian [18]. It largely correlates with the 94-32 cluster and M2 subtype according to multilocus variable-number tandem-repeat analysis (MLVA) and mycobacterial interspersed repetitive unit (MIRU) typing, respectively [19,20]. The members of the population are characterized by a high level of drug resistance and comprise about one-fourth of the pathogen population in Uzbekistan, Tajikistan, Kyrgyzstan, and Kazakhstan [21,22,23]. Additionally, these strains distributed in Russia and other former Soviet Republics [7]. It should be noted, that this population is heterogeneous and includes at least two large clades: CladeA and the previously mentioned CAO. CladeA strains are prevalent in Russia. In turn, CAO isolates are not often identified in Russia and are usually associated with the spread of resistant TB forms in the former Soviet Central Asia [7,21].

In the current study, we examine genetic and phenotypic characteristics of the Rostov strain of *M. tuberculosis* belonging to the CAO clade of the Beijing genotype. This strain was attributed to the pre-extensively drug-resistant (XDR) tuberculosis group. The growth rate and virulence for mice of the Rostov strain were compared with the same characteristics of the *M. tuberculosis* H37Rv strain.

## 2. Results

### 2.1. Genetic and Phenotypic Characteristics of the Strain

The Rostov strain of *Mycobacterium tuberculosis* was isolated in the South Federal District of Russia in 2013 from a patient with pulmonary tuberculosis.

The Beijing genotype (SIT1) was confirmed by spoligotyping. The assignment of the strain to a CAO clade of the Central Asian and Russian Beijing population (Beijing 94-32 cluster) was revealed by PCR assays as in [18,23], respectively. The next generation sequencing analysis on Ion Torrent additionally confirmed that the strain belongs to the CAO clade of the Beijing genotype and carries all previously described CAO-specific single nucleotide polymorphisms (SNPs) [21]. 24-locus MIRU-variable-number tandem-repeat (VNTR) typing scheme revealed 223325153533424682254423 profile which is designated 9358-25 in the international MIRU-VNTRplus database. This profile is closest to the 94-32 and has two different loci, as shown in Figure S1.

According to the drug susceptibility testing and genome analysis, the strain belonged to pre-XDR tuberculosis and was resistant to streptomycin, isoniazid, rifampicin, ethambutol, kanamycin, amikacin, and capreomycin, as shown in Table 1. Additionally, a putative compensatory mutation in the *rpoC* gene (g764363a; G332S) was revealed.

**Figure 1:**
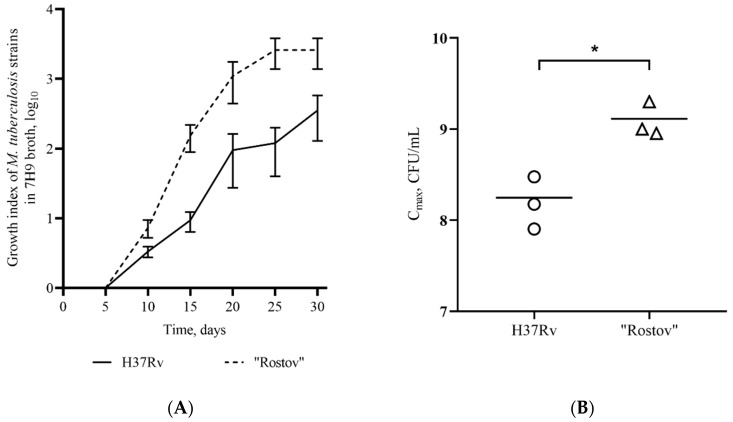
Growth of H37Rv and Rostov strains of *M. tuberculosis* in 7H9 broth. (**A**)—Growth index (calculated by the colony-forming unit (CFU) at each time point divided by the CFU at initial time point); (**B**)—Comparison of *C*_max_ (a maximum point on the growth curve); *—values of *p* < 0.05.

To compare the growth rate between the Rostov and H37Rv strains we determined a growth index and *C*_max_ value, as shown in Figure 1. Growth index reflects that the Rostov strain grew faster than the H37Rv strain throughout the experimental period (*p* < 0.05), as shown in Figure 1A. The Rostov strain showed a higher *C*_max_ than the H37Rv strain (*p* < 0.05), as shown in Figure 1B. *C*_max_ for the Rostov strain was reached on the 25th day, whilst *C*_max_ for the H37Rv strain was reached on the 30th day.

### 2.2. Mice Survival Rate and Bodyweight Dynamic

**Figure 2:**
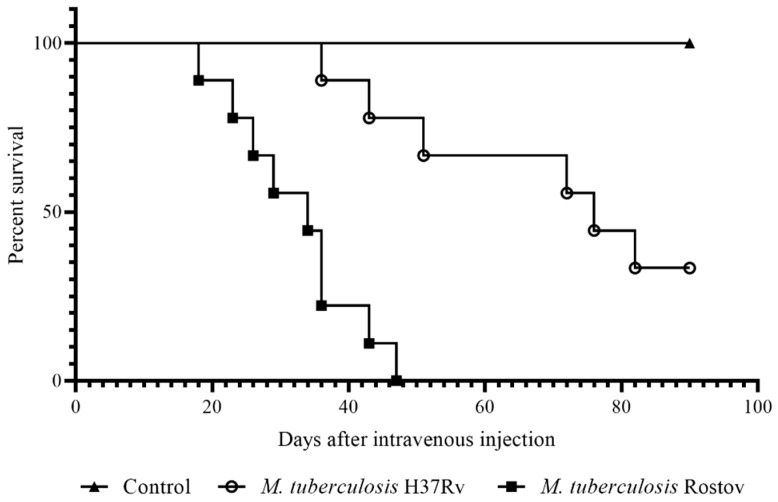
Comparison of the survival curves of C57BL/6 mice infected by *M. tuberculosis* strains. Data were analyzed by the Gehan–Breslow–Wilcoxon test. The value of *p* < 0.05 was taken as statistically significant.

**Figure 3:**
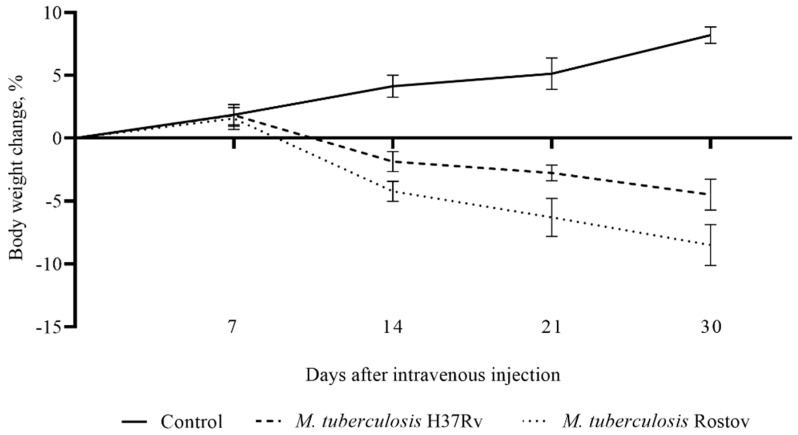
Comparison of the weight changes curves of C57BL/6 mice infected by *M. tuberculosis* strains. The value of *p* < 0.05 was taken as statistically significant.

The model of *M. tuberculosis* infection of C57BL/6 mice was used for the comparison of the virulence of the Rostov clinical strain and the reference virulent strain H37Rv. Animals were intravenously injected with 5 × 10^6^ CFU/mice of each strain (nine mice per strain, *n* = 18). Additionally, as a negative control, a group of uninfected animals (*n* = 9) was used. The patterns of animal survival were observed from the first to 90 days post-infection (p.i.). In each group of mice, weight control was performed. Figure 2 shows that mice infected with the Rostov and H37Rv strains started to die after 18 and 36 days of infection, respectively. All mice of the group infected with the Rostov strain succumbed to death within a 47-day period, while ~56% of mice infected by the H37Rv strain survived within a 90-day p.i. period. Mice weight analysis showed irreversible and severe depletion of animals infected with the clinical Rostov strain compared to animals infected with the laboratory H37Rv strain, as shown in Figure 3.

### 2.3. Investigation of Tuberculosis Process on the 30th Day of Infection

To further define, the virulence of the studied strains, we investigated the tuberculosis process in the C57BL/6 mice models on the 30th day after pathogen injection, when all mice infected by the H37Rv strain were alive, and about 50% of mice infected by the Rostov strain were dead. The pathological processes provided by two *M. tuberculosis* strains were very different. Animal appearance after infection by the H37Rv strain was characterized by mild depletion and smooth fur, but after infection by the Rostov strain—by extremely emaciated and “ruffled” fur. The differences in survival times were associated with differences in the macroscopic appearance of lungs and liver harvested on the 30th day of infection, when more than 50% of mice infected with the Rostov strain were dead. It was shown that the lungs of mice infected by the Rostov strain were different from those in the H37Rv-infected mice, which appeared in increased lungs volume, intensively hyperemic, and no visible nodules; in turn, the lungs of mice infected by the H37Rv strain were pale pink colored with pale mass inclusions. The similar picture was obtained in the liver: Rostov-infected mice livers were dark brown with multiple nodules and the fatty degeneration was visible, while the livers from H37Rv-infected mice were smooth, dark brown, and normal volume, as shown in Table 2.

**Figure 4:**
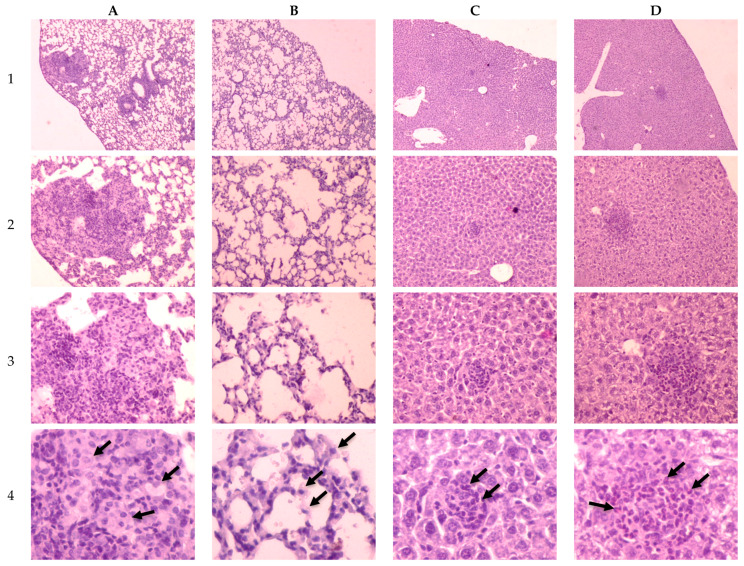
Histology of lungs and livers of C57Bl/6 mice on the 30th day after intravenous inoculation by the *M. tuberculosis* strains H37Rv (**A**—lungs, **C**—liver) and Rostov (**B**—lungs, **D**—liver). 1, 2, 3 and 4—×4, ×10, ×20 and ×40 magnification, respectively. The arrow indicates the specific mice cells (**A**4, **B**4—macrophages; **C**4—lymphocytes; **D**4—polymorphonuclear leukocytes).

The histological investigation of the C57Bl/6 mice infected intravenously by the H37Rv strain of *M. tuberculosis* at a dose of 5 × 10^6^ CFU/animal showed a typical picture for TB mice models in the lungs: the small granulomas composed of numerous macrophages with abundant cytoplasm form; there are some lymphocytes between macrophages; dense perivascular lymphocytic infiltrates form in the lungs in addition to granulomas, as shown in Figure 4A. A single infiltrate consisting of few lymphocytes was found in histological sections of the liver, as shown in Figure 4C.

In contrast, when the C57Bl/6 mice were infected by the Rostov strain of *M. tuberculosis,* the pattern of pathological changes was different. Diffuse thickening of the alveolar septum due to an increased number of macrophages occurred in certain parts of the lungs. Lymphocytic infiltrates were not observed, as shown in Figure 4B. Microscopy of histological sections of the liver showed the presence of nodules consisting of focal cell accumulations in the parenchyma. Cellular infiltrates are composed of few typical macrophages and a large number of polymorphonuclear leukocytes, that indicate intensive pathogen multiplication and the increased development of a pathological inflammatory process, as shown in Figure 4D.

**Figure 5:**
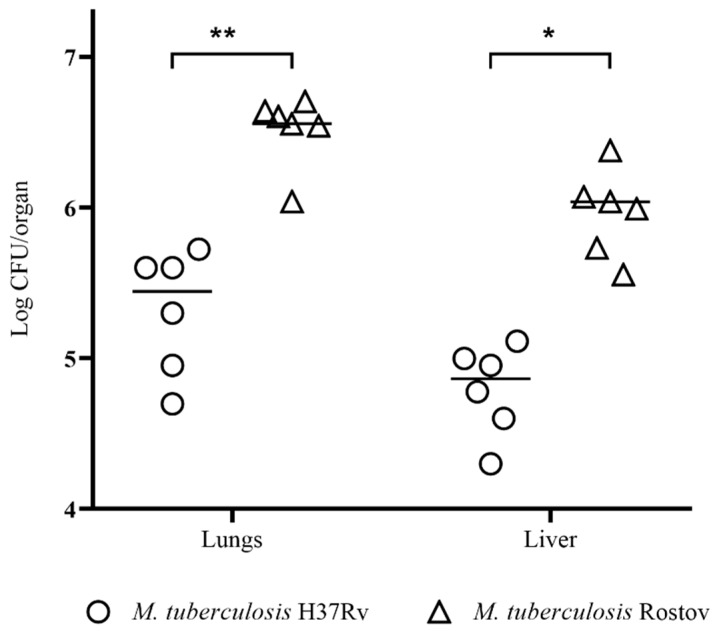
*M. tuberculosis* cells loads of the C57Bl/6 mice lungs and liver on the 30th day after the inoculation of bacteria; *—values of *p* ≤ 0.05; **—values of *p* ≤ 0.01.

Bacterial load in the lungs and liver of mice infected with the Rostov and H37Rv strains was measured on day 30 p.i. According to the data presented in Figure 5, the clinical Rostov strain more actively proliferate in the parenchymatous organs of experimental animals than the H37Rv strains. The overall bacterial load in the lungs was higher than in the liver for both strains.

## 3. Discussion

In order to better understand the virulence properties of CAO strains, we focused on the clinical Rostov strain belonging to the clade. The strain was resistant to seven antituberculosis drugs and contained well-known resistance-associated mutations, as shown in Table 1. Resistance to OFX was not detected for the strain that correlates with a study of Merker et al. [21] in which the frequency of resistance to fluoroquinolones was low among CAO isolates. Besides the drug resistance-associated mutations, the strain carried a compensatory mutation in the *rpoC* gene (g764363a; G332S), which was previously described [7,24]. We suggest that this mutation could affect the fitness and lead to an increased growth rate of the strain compared to the reference H37Rv strain shown in Figure 1, in contrast with data published for 3 strains of lineage 2, that had decreased growth rate compared to the same reference strain [25].

According to 24-locus MIRU-VNTR typing *M. tuberculosis,* Rostov belonged to the 9358-25 cluster and differed from the 94-32 cluster by two loci, as shown in Figure S1. Although this cluster was not described earlier, the phylogenetic analysis using the MIRU-VNTR-plus database revealed a clusterization with the 94-32 type and according to MIRU typing, it belongs to the M2 cluster that is specific to Central Asia population [18].

Analysis of cluster-specific SNPs revealed one significant point mutation (a2321369g; N105D) in the *Rv2063a* (MazF7) gene related to virulence, detoxification and adaptation category according to Mycobrowser database (https://mycobrowser.epfl.ch/) and Forrellad et al. [26]. The second specific SNP was identified in the *fadE29* gene resulting in an amino acid substitution Ile288Val. Such substitution did not provide the significant changes in protein structure, accordingly to BLOSUM62 Matrix [27] (Table S1). It was reported previously that the MazEF toxin–antitoxin system is very important for stress adaptation, drug tolerance, and virulence of *M. tuberculosis*, and required for persistence in vitro. The deletion of MazF reduced the pathogen virulence for guinea pigs and decreased the bacterial load in organs [28]. All other polymorphisms presented in the Table S1 are not specific for the CAO clade, but their role is likely to be important for successful spread of the Beijing genotype in the world.

Survival studies showed that mice infected with Rostov strain succumbed to death within 18–47 days p.i., whereas a large proportion of mice infected with H37Rv maintained viability up to 90 days p.i., as shown in Figure 2. Similar mortality rates were detected for Beijing *M. tuberculosis* strains; conversely, the strains belonging to other *M. tuberculosis* families—Canetti, Haarlem and Somali clades—displayed intermediate or low virulence according to Lopez et al. [9].

Analysis of the specific pulmonary lesions in mice with experimental tuberculosis on day 30 showed that both strains had characteristic pathogenic properties, i.e., were able to cause the tuberculosis process, but patterns of pathological changes in lungs and livers were different for two strains, as shown in Figure 4. Our results are in agreement with Ribeiro et al. [29], according to which the H37Rv strain had the least virulent properties with respect to the Beijing genotype. The obtained data indicate that infection of mice with the clinical Rostov strain of *M. tuberculosis* leads to changes in the lungs. These changes consist of a small increase in the number of macrophages in some interalveolar septums. At the same time, macrophages have a relatively narrow cytoplasm, in contrast to wide-plasma macrophages that infiltrate lung tissue when mice are infected with the H37Rv strain of *M. tuberculosis*. The absence of pulmonary infiltrates in mice infected with the Rostov strain may indicate that this strain did not activate the host defense mechanisms, compared with the response to the infection caused by the H37Rv strain, as shown in Figure 4. 

In conclusion, our study showed that pre-XDR Rostov strain belonging to the CAO clade of *M. tuberculosis* Beijing genotype is characterized by high virulence for C57Bl/6 mice when compared with the laboratory H37Rv strain. We propose that characteristic alterations of the CAO clade favor the selection of highly virulent bacteria.

## 4. Materials and Methods

### 4.1. M. tuberculosis Strains

The Rostov strain of *M. tuberculosis* was initially isolated from a clinical sample of sputum collected from a 35-year-old man hospitalized in the South Federal District of Russia in 2013 and deposed into the State Collection of Pathogenic Microorganisms “SCPM-Obolensk” (ID B-7601). The virulent laboratory H37Rv strain of *M. tuberculosis* was obtained from the “SCPM-Obolensk” collection (ID B-4825). 

Frozen stocks of bacterial cells (1 × 10^5^ CFU) were inoculated into 30 mL Middlebrook 7H9 broth with OADC supplement (BD, Franklin Lakes, NJ, USA), and 0.05% Tween 80, in three biological replicates, incubated at 37 °C under static conditions (i.e., without agitation) in flask cell culture 250 mL (Greiner AG, Kremsmünster, Austria) for 30 days to estimate the growth rate. Every five days the aliquots of 0.1 mL were taken for CFU enumeration by plating the serial 10-fold dilutions in triplicates onto Middlebrook 7H11 agar (BD, Franklin Lakes, NJ, USA) enriched with OADC. Bacterial colonies were counted on the plates after incubation for three weeks at 37 °C. To compare the growth rate amongst strains, we determined a growth index, calculated from the log_10_ of the number of CFU at each time point divided by the log_10_ of the number of CFU at the initial time point. Additionally, we used *C*_max_ to compare the growth of the strains. This index means the peak point on the bacterial growth curve [25].

### 4.2. Antibacterial Susceptibility

*M. tuberculosis* drug susceptibility testing (DST) of the Rostov strain to isoniazid 1.0 mg/L (INH), rifampin 40.0 mg/L (RIF), streptomycin 10.0 mg/L (STR), ethambutol 5.0 mg/L (EMB), amikacin 30.0 mg/L (AMK), kanamycin 30.0 mg/L (KAN), capreomycin 30.0 mg/L (CAP), and ofloxacin 3.0 mg/L (OFX) was carried out using the method of absolute concentrations on solid Lowenstein–Jensen medium [30]. In addition, DST was performed by the BACTEC MGIT 960 system (BD, Sparks, MD, USA) according to the manufacturer’s instructions. 

### 4.3. Genomic Analysis

Genomic DNA was isolated from the Rostov strain of *M. tuberculosis* using a standard extraction method [31].

Spoligotyping and 24 MIRU-VNTR typing were performed as described in references [32,33], respectively. Verification of the presence of SNP in the *sigA* gene and CAO-specific IS*6110* insertion in the *Rv1359-Rv1360* intergenic region was performed by PCR as described previously [18,34].

Whole genome sequencing was performed on Ion Torrent PGM (Life Technologies, Camarillo, CA, USA) with Ion 318 chip and Ion PGM™ Sequencing 200 Kit v2 (Life Technologies, Camarillo, CA, USA). Raw sequence data were submitted to the NCBI under the project PRJNA269675. The genome was assembled using Newbler GS de novo assembler 2.5 (Roche, Branford, CT, USA) with standard parameters for Ion technology. SNPs were detected with Snippy v.4.3.6 (https://github.com/tseemann/snippy) pipeline with a minimum coverage depth of 10 and an alternate fraction of 0.9. A comprehensive list of drug-resistance mutations to first- and second-line drugs was used to determine genetic resistance of the strain [35]. Functional categories and virulence factors were defined according to Mycobrowser (https://mycobrowser.epfl.ch/) and [27].

### 4.4. Bioethical Requirements

All animal experiments were carried out in full accordance with the European Convention for the Protection of Vertebrate Animals, used for experimental and other scientific purposes (Directive 2010/63/EU of the European Parliament and of the Council of 22 September 2010 on the protection of animals used for scientific purposes), and the requirements of Sanitary Regulations 1.3.2322-08 “Safety of work with microorganisms of the III-IV pathogenicity groups and pathogens of parasitic diseases”, and Veterinary Protocol No. VP-2016/8 were approved by bioethics of the State Research Center for Applied Microbiology and Biotechnology.

### 4.5. Mice Infection 

Specified pathogen-free female C57BL/6 mice (*n* = 51) were obtained from Shemyakin and Ovchinnikov Institute of Bioorganic Chemistry RAS (Moscow, Russia). All mice were used at 7–8 weeks of age and 20–22 g in weight. Randomization was used to allocate three experimental groups: control, *M. tuberculosis* H37Rv, and *M. tuberculosis* Rostov.

*M. tuberculosis* strains were grown to mid-logarithmic phase (OD_600_ = 1.0), cells were collected by centrifugation, and washed with PBS containing 0.05% Tween-80. Mice were intravenously injected into the lateral tail vein with 5 × 10^6^ CFU/mice (in 0.1 mL of 0.9% NaCl) of the H37Rv strain and of the Rostov strain. All animals were weighed each day after infection. Animals were observed for 90 days; the physical appearance and behavior of animals were estimated; the daily animal weight loss and mortality were calculated.

On the 30th day after *M. tuberculosis* injection, six mice were euthanized by CO_2_ gas in each experimental group. Lungs and livers tissues were examined for mycobacterial load and pathology. The *M. tuberculosis* bacillary burden in lungs and livers was counted by homogenates plating onto Middlebrook 7H11 agar. Some samples of lungs and livers were fixed in 10% formalin (BioChem-NN, Nizhny Novgorod, Russia), graded concentrations of ethanol and butanol were used for dehydration, embedded in paraffin, and serial sections (5 µm width) were prepared with the Ultracut microtome (Reichert-Jung, Bensheim, Germany). Sections were deparaffinated and stained with hematoxylin and eosin. All slides were examined with a Nikon Eclipse 80i microscope and a Nikon DS-U2digital camera (Nikon, Tokyo, Japan).

### 4.6. Statistical Methods

Analysis of data was conducted using GraphPad Prism version 8.0.1 for Windows (GraphPad Software, La Jolla, CA, USA, www.graphpad.com). Statistical analysis between groups was performed using the analysis of variance (ANOVA) test. Survival data were analyzed using the Gehan–Breslow–Wilcoxon test. The growth index and *C*_max_ were compared amongst the H37Rv and Rostov strains using the unpaired t-test at each time point. A value of *p* < 0.05 was considered significant.

## Figures and Tables

**Table 1:** MIC distribution of the Rostov strain of *Mycobacterium tuberculosis* for antibiotics and drug resistance markers.

Antibiotic	MIC, mg/L	Interpretation	Drug Resistance Marker
Isoniazid (INH)	>1	R	KatG (S315T)
Rifampicin (RIF)	>40	R	RpoB (S450L)
Streptomycin (STR)	>10	R	RpsL (K43R)
Ethambutol (EMB)	>5	R	EmbB (M306V)
Amikacin (AMK)	>30	R	*rrs* (a1401g)
Kanamycin (KAN)	>30	R	*rrs* (a1401g)
Capreomycin (CAP)	>30	R	*rrs* (a1401g)
Ofloxacin (OFX)	≤3	S	-

Note: MIC—minimal inhibitory concentration; R—resistance; S—sensitivity.

**Table 2:** Comparative characterization of the mortality, animal appearance and morphological description of internal organs of C57Bl/6 mice infected by the H37Rv and Rostov strains of *M. tuberculosis*.

Strain	The Mortality Rate on 30th p.i. Day, %	Animal Appearance	Lungs	Liver
H37Rv	0	Mild depletion, smooth fur	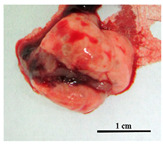 Pale pink colored with pale mass inclusions	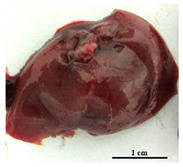 Smooth, intense brown, normal volume
Rostov	~50%	Extreme emaciated, hunched posture, “ruffled” fur, reduced movement	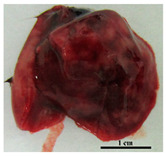 Intensively hyperemic, no visible nodules	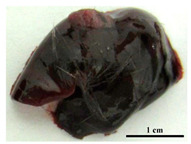 Dark brown with multiple nodules, fatty degeneration

## Supplementary Materials

The following are available online at https://www.mdpi.com/2076-0817/9/5/335/s1, Figure S1. MIRU-VNTRplus cluster analysis of *M. tuberculosis* Rostov strain, Table S1. Sublineage-specific nsSNPs in the virulence genes.
