# Supplementary figures and images for: Genotyping, Assessment of Virulence and Antibacterial Resistance of the Rostov Strain of Mycobacterium tuberculosis Attributed to the Central Asia Outbreak Clade
Source: Pathogens. 2020 Apr 30;9(5):335. doi: 10.3390/pathogens9050335 (PMC7281402; doi:10.3390/pathogens9050335)

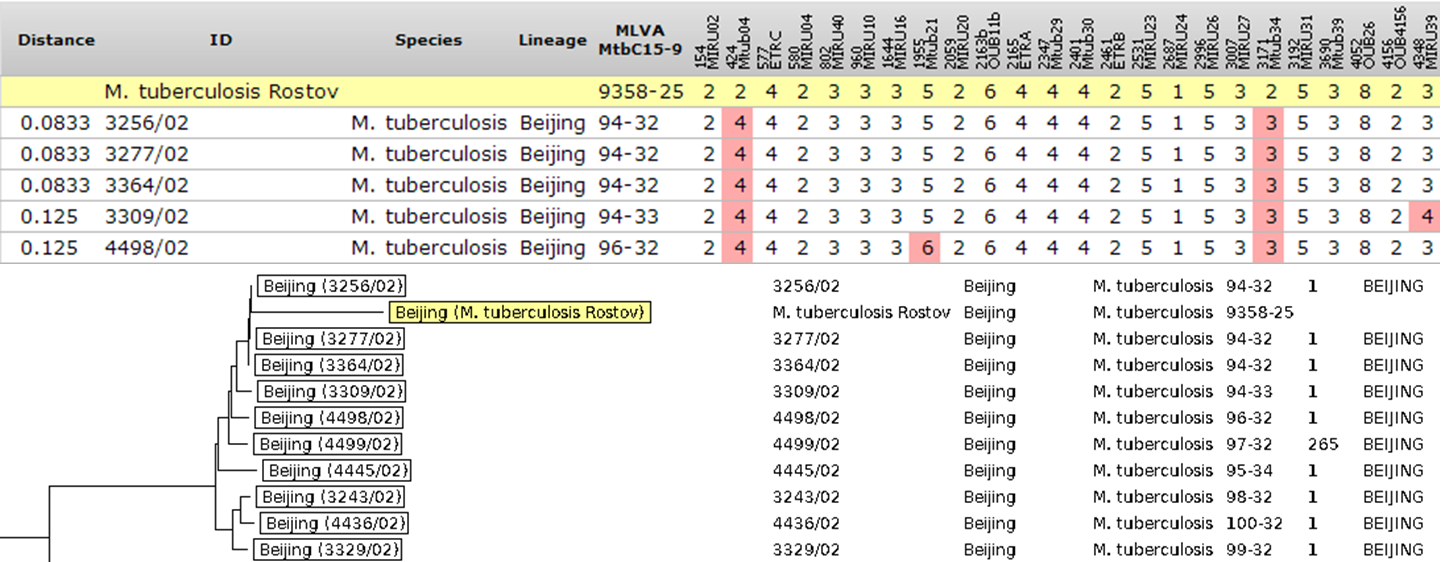

Supplement: Supplementary file 1 [file pathogens-09-00335-s001.zip › pathogens-746320-supplementary/Figure S1.tif]
